# Perspectives of the Association of Academic Neonatology Division Directors on the American Board of Pediatrics recommendations for training in neonatal-perinatal medicine

**DOI:** 10.1038/s41372-026-02806-7

**Published:** 2026-07-08

**Authors:** P. J. McNamara, D. O’Reilly, M. Good

**Affiliations:** 1 University of Iowa Stead Family Children’s Hospital, Iowa City, IO USA; 2 University of Vermont College of Medicine, Burlington, VT USA; 3 University of North Carolina School of Medicine, Chapel Hill, NC USA

**Keywords:** Health occupations, Scientific community

## Abstract

Recommendations from the American Board of Pediatrics propose a two-year fellowship for Neonatal-Perinatal Medicine. A survey of members of the Association of Academic Neonatology Division Directors was conducted to gather perspectives on the recommendations. A minority of respondents were supportive of 2-year fellowships. Although there may be some financial benefit by transitioning to a faculty salary earlier, risk of burnout, attrition of the academic and physician scientist pipeline, and curtailing innovation and discovery were cited as major concerns. Division directors are supportive of enhancing clinical exposure, transition to competency-based assessment, and the creation of trainee pathways within 3-year fellowships.

The American Board of Pediatrics (ABP) has proposed a 2-year clinical fellowship pathway consisting of 18 training blocks with no mandatory scholarly work product. This compresses and increases required clinical training by roughly 50% (from 12 months to 18 blocks) over a shorter duration. A recent expert statement raised concerns that the compressed clinical timeline would simultaneously decrease formal didactic education by one-third, meaning the program demands more from fellows clinically while delivering less educationally [1]. A survey representing 110 of the 111 national neonatal-perinatal medicine (NPM) fellowship programs revealed that 76% of program directors oppose the 2-year pathway. Whether these perspectives are shared by Neonatology division directors is unknown.

## Methods

This was a cross-sectional survey designed to understand the perspectives of Neonatology division directors at academic neonatal programs across the United States, on the implications of the recent ABP recommendations for NPM fellowship training. The Association of Academic Division Directors of Neonatology (AANDD) is an independent national collaboration of leaders at academic centers, and membership is defined by the presence of an NPM fellowship or residency program. The content of the survey was designed based on the survey tool disseminated to NPM fellowship program directors, with some additional questions added. The 10-step instrument was designed using the University of Iowa Qualtrics application and evaluated by two members of this writing group (PM, MG). The survey was disseminated via email in May 2026 to the AANDD listserv, which includes 118 members, with a request that each program submit a single response from the division director. One follow-up reminder to complete the survey was sent one week later to maximize the response rate. Our a priori goal was to achieve a response rate of at least 50% (59 respondents) to ensure a representative sample. The frequency of each response was recorded using a Likert scale. Descriptive summary statistics were used to analyze the data.

## Results

In total, 74 (63%) neonatologists completed the survey. A minority of respondents [*n* = 12, (16%)] were supportive of the 2-year fellowship model (Table 1). Few respondents reported a plan to transition to 2-year fellowships [*n* = 4, (5.4%)]. Of note, a majority of respondents were supportive of a separate neonatal residency [*n* = 56, (76%)] and the establishment of an independent department of Neonatology [*n* = 46, (62%)]. Proponents of a 2-year fellowship program reported lower financial burden and elimination of mandatory scholarly activity as strengths of this model; however, the most common response was “no benefit” (Table 2). Challenges of the 2-year model included feasibility of the model, risk of burnout, attrition of the academic neonatologist and physician-scientist pipeline, and disruption of innovation and discovery. There was equipoise over whether applications to neonatal-perinatal fellowships would increase.

**Table 1:** Perspectives of individual division directors of Neonatology at academic medical centers on the recommendations of the American Board of Pediatics.

*Question*	Frequency
*What is your overall level of support for the ABP’s new 2-year clinical only option?*	Strongly Against (*n* = 31) Against (*n* = 28) Neutral (*n* = 3) Support (*n* = 8) Strongly Support (*n* = 4)
*Based on the limited information currently available, what do you envision your program offering:*	Mostly 3-year option (*n* = 28) Mostly 2-year option (*n* = 4) Mixture of 2- and 3-year options (*n* = 27) Unsure (*n* = 15)
*How many months or 4-week blocks of clinical time does your program currently do in three years?*	<6 months (*n* = 3) 6–8 months (*n* = 10) ≥9 months (*n* = 61)
*How frequently do fellows currently take call each month?*	<2 nights (*n* = 2) 2–4 nights (*n* = 54) ≥5 nights (*n* = 18)
*How do you expect call frequency to change after the transition?*	Decreased number of shifts (*n* = 6) Increased number of shifts (*n* = 18) Stay the same (*n* = 20) Unsure (*n* = 29)
*What is your current opinion: Neonatology should separate from general pediatrics and form its own Department of Neonatology*.	Strongly Agree (*n* = 23) Agree (*n* = 23) Disagree (*n* = 18) Strongly Disagree (*n* = 10)
*What is your current opinion: Neonatology should have a training pathway that is distinct from general pediatrics and other pediatric subspecialties*.	Strongly Agree (*n *= 34) Agree (*n* = 22) Disagree (*n* = 13) Strongly Disagree (*n* = 5)

**Table 2:** Perspectives on the advantages and challenges of the recommendations of the American Board of Pediatics.

What are potential ADVANTAGES of a 2-year pathway?	- More applications for neonatal-perinatal fellowships (*n* = 10) - Lower financial burden (*n* = 19) - More neonatologist workforce (*n* = 4) - No benefits (*n* = 24) - Avoidance of mandatory research expectation (*n* = 11) - More focused training for community setting (*n* = 5)
Select comments	“I feel it may attract more resident and fellow applications” “Not everyone appreciates research and the requirement is probably unevenly applied in the current model. The 2 year will also allow quicker movement to improved pay.” “It may broaden the appeal of neonatology to pediatric residents with financial hardships.” “A shorter training pathway may attract some trainees who are deterred by the length of a traditional 3-year program, particularly those seeking to enter the workforce more quickly.”
What are potential CHALLENGES of the 2-year pathway?	- Feasibility of completing 18 clinical blocks in 2-year fellowship is questionable (*n* = 21) - Lack of research training will decrease innovation and discovery in the field (*n* = 26) - Trainee education will be compromised (*n* = 18) - Trainee wellness will suffer with increased risk of burnout (*n* = 12) - Financial stability of the model (*n* = 6) - Attrition of academic neonatologist workforce (*n* = 29) - Abolition of physician scientist pipeline (*n* = 11) - Loss of training in quality improvement and patient safety methodology (*n* = 6) - Loss of candidates for neonatal-perinatal fellowships (*n* = 12) - Eligibility of graduates of 2-year programs to work at academic medical centers (*n* = 8)
Select comments	“I will never hire a neonatologist with 2 years of training” “This is the end of academic neonatology” “The spirit of innovation and discovery will be lost” “Why are individuals with no formal training in Neonatology driving our future.” “We are creating a race to the bottom” “Model not feasible as trainees unionized”

In this survey, which is representative of the opinions of a high proportion of AANDD, responses were predominantly negative or strongly negative, with limited support for the ABP recommendations regarding a two-year fellowship, and frequent conditional acceptance contingent on implementation details and flexibility. The field of Neonatology has witnessed major advancements over the past thirty years, with increased survival and improved outcomes for extremely preterm infants and patients with complex neonatal illness [2]. The rationale for these improvements is likely to be multifactorial; however, an investment in science, discovery, and innovation is foundational to these successes. Exposure to scientific investigation and the expectation to engage in scholarly activity have been the catalyst for many trainees to pursue a career in academic neonatology. Elimination of research and scholarly work is expected to diminish critical thinking, quality improvement skills, and evidence-based practice, threatening not only the physician-scientist pipeline and future innovation but also the development of clinicians with knowledge and leadership skills to care for complex infants in acute clinical scenarios.

Neonatology is a high-stakes environment which requires mastery of advanced therapies (e.g., ECMO, therapeutic hypothermia, delivery room resuscitation at the limits of viability). Recent decreases in ICU exposure during general pediatric residencies, which have not yet been fully realized, mean that incoming fellows often start their training with minimal real-life procedural and patient-care experience in the NICU. Therefore, the recommendation to increase the number of clinical blocks and the transition to competency-based assessment are important steps forward, which most AANDD members support. Many respondents, however, believe that a compressed 2-year timeframe is insufficient for achieving full clinical competency, especially given reduced residency exposure and limited procedural opportunities. While this model may increase the number of practicing clinicians, most respondents anticipate a decline in academic neonatologists, educators, and physician-scientists. In addition, compressing 18 clinical blocks (72 weeks) into 22–24 months would require fellows to log ~3500 training hours annually [1], exceeding the expectations of a neonatologist in both academic and private practice settings [3, 4]. This equates to an average of 78–80 h per week, nearly doubling the clinical workload and thereby threatening the sustainability of the training and violating ACGME duty-hour requirements.

While shorter training may reduce debt burden and attract fellowship applicants, it may create disincentives for academic careers and raises concerns about funding for extended training pathways. Significant ambiguity remains regarding competency assessment, program structure, match processes, and financial sustainability of supporting the optional third year for research track, complicating the planning and execution of the ABP proposal. Shortening the standard training pathway may financially incentivize hospital administrations to cut program length based on fiscal factors rather than educational competency. On the contrary, perceived advantages of the proposed model include the potential to increase recruitment into neonatology, reduce the financial burden, enable earlier transition to attending-level salaries, accelerate the expansion of the clinical workforce, and offer a more appealing pathway for trainees seeking purely clinical career paths [5, 6].

In summary, the proposed 2-year clinical pathway is widely perceived by most division directors as prioritizing workforce efficiency and financial considerations at the potential expense of clinical and procedural competence, trainee burnout, and the long-term sustainability of academic neonatology. One additional consideration is the growth of additional subspecialty fellowships in Neonatology (e.g., neonatal hemodynamics, neurocritical care). There is an urgent need to develop optimal trainee pathways in neonatal-perinatal medicine that support a variety of career options, including academic clinical neonatologists, physician-scientists, and private- and community-based neonatologists.
